# Alcohol and Violence in the Lives of Gang Members

**Published:** 2001

**Authors:** Geoffrey P. Hunt, Karen Joe Laidler

**Affiliations:** Geoffrey P. Hunt, Ph.D., is a senior scientist at the Institute for Scientific Analysis, Alameda, California. Karen Joe Laidler, Ph.D., is an associate professor of sociology at the University of Hong Kong, Hong Kong, SAR of China

**Keywords:** AODR (alcohol or other drug [AOD] related) violence, gang, youth culture, juvenile delinquency, AOD use as a form of socializing, gender identity, male, group behavior, peer pressure, attitude toward AOD

## Abstract

Life within a gang includes two endemic features: violence and alcohol. Yet, to date, most researchers studying gang behavior have focused on violence and its relationship to illicit drugs, largely neglecting the importance of alcohol in gang life. Because alcohol is an integral and regular part of socializing within gang life, drinking works as a social lubricant, or social glue, to maintain not only the cohesion and social solidarity of the gang, but also to affirm masculinity and male togetherness. In addition to its role as a cohesive mechanism, particular drinking styles within gangs may operate, as with other social groups, as a mechanism to maintain group boundaries, thereby demarcating one gang from another. Other examples of internal gang violent activities associated with drinking include fighting between members because of rivalries, tensions, or notions of honor or respect. At a more symbolic level, drinking is associated with two important ritual events in gang life: initiation, or “jumping in,” and funerals. By better understanding the link between drinking and violence among youth gangs, steps can be taken to determine the social processes that occur in the development of violent behavior after drinking.

Social commentators have had a longstanding interest in and preoccupation with youth groups and violence. Even in Victorian England in the late 19th century, commentators were preoccupied with the apparent escalation in disorder among young violent working-class youth gangs in the industrial cities of Birmingham, Liverpool, and Manchester (Davies 1999, p. 72). In the United States, the first major social science study of youth gangs dates back to the 1920s with the publication of Thrasher’s monumental study of 1,313 Chicago gangs (Thrasher 1927). Since then, the interest in gangs from a research perspective, as well as from a media and criminal justice standpoint, has periodically surfaced. The latest wave and, without a doubt, the peak of public concern over gangs began in the mid-1980s and has continued to the present. Spurred on by the media, law enforcement officials started to take a renewed interest in gangs because of their involvement in drug use and drug sales and because of the belief that gangs were the breeding ground for serious delinquency. The development of the drug trade in the 1980s signaled a transformation from the idea of gangs as “transitory adolescent social networks to nascent criminal organizations” (Fagan 1990, p. 183). Gang researchers began to argue that a new type of youth gang had developed which was qualitatively different from gangs in earlier periods. The new type of gang possessed a “raison d’etre” no longer based on cultural factors, such as attachment to a neighborhood, and notions of individual as well as collective honor, but, instead, centered on the economic rationale of profit making (Goldstein 1989; Skolnick et al. 1989).

In spite of the importance of drugs within the social life of gangs, the recent preoccupation of research on illicit drugs has overshadowed the importance of drinking within youth gangs and its possible relationship to violent behavior. To date, little research has been done specifically on the role of alcohol within the social life of gangs, and consequently, it is a relatively unexplored area. The available research data on the role of alcohol in youth gangs has been, to paraphrase Dwight Heath (1975), a “felicitous by-product” of other interests. This is in spite of the fact, as Fagan (1993) has noted, that alcohol is still the most widely used substance by both gang and nongang youth. This absence of research on the role of alcohol within gang life is particularly striking in the available work on the violent behavior of gang members.

Over the past decade, researchers and public health officials have become increasingly concerned with the increased involvement of youth in violent crime. The juvenile male arrest rate for violent crime offenses increased steadily during the first half of the 1990s, peaking in 1994 with a rate of 880, and then beginning to drop, with an arrest rate of 545 in 1999. Among females, the juvenile arrest rate for violent crime also has risen, peaking in 1995 at 158 and then leveling off at 122 in 1999 (*a*Office of Juvenile Justice and Delinquency Prevention [OJJDP] 2000*a*). Within this growing concern, considerable attention has focused on youth gangs as a key factor. Currently, more than 90 percent of the Nation’s largest cities report youth gang problems, and police estimates now put the number of gangs at 28,700 and the number of gang members at approximately 780,200 (OJJDP 2000*b*). As a result, public concern about the involvement of young people in gang activity, and the perceived violence associated with this lifestyle, has soared.

Violence is endemic to gang life. As Sanchez-Jankowski (1991) has noted, it is the “currency of life” within gangs, so much so that it can be taken as normative behavior (Collins 1988). Violence within gang life includes both intragang violence—for example, ritualistically violent initiations (Vigil and Long 1990)—and intergang violence—for example, turf battles (Sanchez-Jankowski 1991). In order to explain the daily occurrence of violence within the social life of gangs, both researchers and criminal justice officials have tended to focus their recent attention on the role of illicit drugs as a crucial explanatory factor (Fagan 1989; Klein and Maxson 1989; Moore 1991; Reiss and Roth 1993). This preoccupation with gangs and drug-related violence has tended to overshadow the significant role of alcohol in gang life and its possible relationship with violent behavior.

In the same way that violent behavior is a common currency within gang life, so also is drinking. Although few researchers have looked specifically at the role of alcohol within the social life of gangs, some gang researchers have noted that drinking is a major component of the social life of gangs and a commonplace activity (Fagan 1993; Hagedorn 1988; Moore 1991; Padilla 1992; Vigil and Long 1990). Therefore, given the extent to which drinking has been implicated in a wide range of interpersonal violence (Parker 1995; Pernanen 1991; White et al. 1993) and the extent to which alcohol problems have been found to occur disproportionately among both juveniles and adults who report violent behaviors (Collins 1986; Jacob and Leonard 1994), an examination of the possible relationship between alcohol and violence in gang life is an important area of investigation. Such an investigation can highlight both the extent to which drinking is present in “violent prone situations” within gang life (Steadman 1982) and the way in which drinking and normative violence are everyday features of gang life. This article examines the arenas in which drinking is present in the life of gangs and reviews existing literature on the situations and contexts in which drinking is associated with violent behavior.

## Alcohol and Gang Life

To understand the role of alcohol in the lives of gang members, we must begin our analysis by considering the characteristics and dynamics of street life. As many researchers have noted, being on the streets is a natural and legitimized social arena for many working-class, minority male adolescents. For many of these young men, life is “neither the workplace nor the school; it is the street” (Messerschmidt 1993, p. 102). Life on the streets is governed by rules of masculinity, in which notions of honor, respect, and status afford outlets for expressing and defending one’s masculinity. The entry to life on the street is through a street gang. The gang epitomizes masculinity and ensures male bonding. Once in the gang, young men gain status and respect through their ability both to assert themselves by being “street smart” and to defend their fellow gang members (i.e., homeboys) by “being down,” or being ready to come to the aid of fellow gang members should they need assistance. Working-class, minority gang members can gain respect through their ability to fight (Anderson 1999). Not only must they be prepared to defend themselves and their fellow gang members, they also must be prepared to defend the reputation of their gang. Given the masculine culture of street life, what role does alcohol play?

### Hanging Around

Gang members spend the majority of their day “hanging around” (Corrigan 1976), or “just chilling,” and typically describe this activity in the very mundane terms of “doing nothing.” Although adults perceive this as a waste of time, the everyday practice of “doing nothing” is often an intense and busy period, and the activities that occur include talking, recounting details from previous events, joking, discussing business, defending one’s honor, maintaining one’s respect, fending off insults, keeping the police at bay, “cruising” around in a car, doing a few deals, defending turf, and getting high. During most of these activities, drinking is endemic, and the consumption of alcohol occurs continually throughout the course of everyday social activities. As with many other social groups, drinking can be said to act as a social “lubricant,” or “social glue,” working to maintain cohesion and group solidarity (Vigil and Long 1990).

### Partying

Partying is a focal event in the life of gang members in which binge drinking is an integral component (Moore 1991; Moore et al. 1978; Vigil 1988).

Partying overlaps with gang members “hanging out,” and gang members “party” both at public dance places, bars, and parks in the neighborhood or at private parties held in hotel rooms or at someone’s home or garage. Sometimes private parties are arranged formally, organized either for celebrations or, on some occasions, for grieving. Like hanging out, partying also operates to maintain and enhance the cohesion of the group (Moore 1991).

### Symbolic Drinking

Drinking works in several symbolic ways in the gang. Because drinking is an integral and regular part of socializing within gang life, as the table illustrates, drinking works as a social lubricant, or social glue, to maintain not only the cohesion and social solidarity of the gang but also to affirm masculinity and male togetherness (Dunning et al. 1988). Comparisons across the different ethnic gangs, however, suggest that drinking affirms masculinity in culturally defined ways. Existing research on Latino gangs suggests that drinking plays a key role in the creation of a “macho” identity. “Machismo” includes demonstrations of strength and toughness as well as “locura” (i.e., acting crazy or wild) (Moore 1991; Padilla 1992; Vigil and Long 1990). As Vigil and Long (1990) have noted, alcohol can work as a “facilitator” in the observance of ritually wild or crazy behavior, especially in violent conflicts with outsiders.

Studies of African-American gang life suggest the construction of a different cultural identity, one in which “the overall street style and the desired approach to projecting an individual’s personal image can be summed up in the word ‘cool’” (Feldman et al. 1985, p. 124; see also Hagedorn 1988; Taylor 1989). In this subculture, occasional drinking is the norm (MacLeod 1987) in both public and private settings. Although the African-American gang members in our sample reported relatively higher alcohol use than the other ethnic groups, the style of drinking and the behavior associated with it stress that intoxicated drinking undermines the “cool” image and is likely to be interpreted as a sign of “being out of control.” In the case of Asians and Pacific Islanders, the available research suggests different attitudes to drinking. On the one hand, Chin (1990) suggests that Chinese gangs frown upon intoxication. On the other hand, other work on both Asian and Pacific Islander and Southeast Asian gangs (Toy 1992; Waldorf et al. 1994) suggests that although drinking is not heavy among these groups, it is nevertheless widespread.

In addition to its role as a cohesive mechanism, particular drinking styles within gangs may operate, as with other social groups (Cohen 1985), as a mechanism to maintain group boundaries, thereby demarcating one gang from another. In this way, particular drinking styles can be seen as similar to other symbolic insignia, including tattoos, dress colors, and codes (Miller 1995).

Other examples of internal gang violent activities associated with drinking include fighting between members because of rivalries, tensions, or notions of honor or respect. Tensions may arise when two gang members or cliques compete for power or status within the gang or when two members compete over the affection of another. After bouts of drinking, these simmering rivalries may erupt, and fighting often occurs. In such cases, as other researchers have noted, alcohol works to create a ritualized context for fighting and violent confrontations, whether physical or verbal (Szwed 1966; Macandrew and Edgerton 1969), in which in-built tensions can be released or disputes settled within a contained arena. Once resolved through alcohol-related violence, the group can maintain its cohesion and unity. In fact, on some occasions, once the conflict has ceased, the antagonists seal their unity by sharing a beer.

At a more manifestly symbolic level, drinking is associated with two important ritual events in gang life. For many gangs, new members are expected to go through an initiation, often referred to as “jumping in.” This induction process, or rite of passage, is important, because it is designed to symbolically test the newcomer’s toughness and his ability to defend himself and withstand physical violence. The ritualized physical testing of potential group members is a common occurrence in many societies and has been described and analyzed by many anthropologists. As Heald (1986) has noted, group initiations are similar to examples of “battleproofing” in military training, in which the new recruits experience a situation of stress that allows them “. . . to develop confidence in their ability to face danger” (Heald 1986, p. 78). Within the gang, as Vigil and Long (1990) have noted, this process can serve “to test member’s toughness and desire for membership . . . and to enhance loyalty to the group” (Vigil and Long, p. 64). Once the initiation has been accomplished and the newcomers accepted, their new status is confirmed by a bout of drinking and getting drunk. The association of alcohol and violent behavior is confirmed. The act of drinking and getting drunk after being “jumped in” also can help to deaden the pain resulting from the violence of the initiation.

Funerals are the second ritual events in gang life in which alcohol and violence are associated. Here, gang members in mourning use alcohol to represent their connectedness to the dead homeboy. They pour alcohol over the grave or leave alcohol at the graveside to symbolize their unity. For example, Campbell describes an incident in which each gang member, during a drinking session, ritually poured alcohol onto the floor prior to drinking from a bottle of rum, which was being passed around: “. . . he pours a little of the rum on the ground in memory of those who are dead or who are in jail” (Campbell 1991, p. 55). After the funeral has taken place, the group may begin heavy drinking. At this time, the gang members may again symbolize their connectedness to the dead by pouring some of their beer or alcohol on the ground. Drinking also may lead the group to decide on taking revenge, either on those responsible for the death of their homeboy or, in some cases, on innocent individuals.

In addition to those violent activities internal to the gang, violence between opposing gangs is another frequent and common activity that is associated with drinking. Different types of activities include the following: violence targeting a member or members of a rival gang, violence against residents of the gang’s own neighborhood, and violence against gangs or residents of another neighborhood (Sanchez-Jankowski 1991). The reasons for such conflict are varied and include such issues as gang members testing others, gang members’ perceptions that they or their territories have been “disrespected,” gang members’ fears that their turfs are under threat, gang members’ attempts to expand their turf, and fighting over the affections of another. In many of these types of external violent activities, drinking prior to the event is common.

Although gang members expressed no overall agreement on the issue of whether alcohol improved their ability to fight, two features were clear. First, confrontations between rival gang members frequently took place while gang members were drinking. Second, in spite of disagreements as to the precise effects of alcohol, many of our respondents admitted that drinking assisted them to develop a sense of “locura” (Vigil and Long 1990), or “pumped them up,” making them ready to fight. In these cases, alcohol works not as the literature would suggest, as an excuse or deviance disavowal mechanism (Heath 1978; MacAndrew and Edgerton 1969), but, instead, as an enabling mechanism.

Thus, alcohol may be perceived as the catalyst for violent incidents, either because drinking was more likely to lead to fighting or because, once drunk, the gang members did not care what happened. Violent confrontation with another group may be senseless and provoked by a drunken fellow homeboy; nevertheless, because of their notions of respect and honor, the members must still defend their homeboy. These violent activities, which are encouraged by the young men drinking, work to bind the group together (Sanders 1994). The identity of the group is continually reinforced by these conflicts with other gangs or with other individuals, while enforcing the gang’s separateness.

While alcohol and group drinking can work to maintain and confirm group cohesion, it also can operate in a divisive manner (Hunt and Satterlee 1986). These internal conflicts occur because honor and respect have been questioned or previous rivalries and tensions have come to the surface. Fighting often occurs because of a supposed slight, an accident, or an unfortunate remark or for paying too much attention to somebody’s girlfriend—any action that might be interpreted as showing disrespect. Fortunately, conflicts within the gang do not appear to lead to long-lasting rifts; once the fighting is over, the group reconvenes. In such cases, alcohol works to create a “time-out” period, or a ritualized context for fighting and violent confrontations, whether physical or verbal (MacAndrew and Edgerton 1969), in which in-built tensions can be released or disputes settled within a contained arena. Once the tensions are resolved through alcohol-related violence, the group can again regain its cohesion and unity.

## SUMMARY

Few researchers have examined the interconnections between two endemic features of gang life: violence and drinking. To date, most gang researchers have focused on violence and its relationship to illicit drugs and have largely neglected the importance of alcohol in gang life. This article provides a brief review of the extent to which drinking is a pervasive feature of gang life and the ways in which drinking connects with different types and settings of violent behavior.

In focusing on drinking and violence among youth gangs, researchers can begin to explore the social processes that occur in the development of violent behavior after drinking (Pernanen 1991). This focus is important, because many researchers (Roizen 1993) have noted that in spite of the “. . . hundreds of studies that have addressed aspects of the relationship between drinking and violence” (Collins 1988, p.108), little is known about alcohol’s role in violent behavior.

## Figures and Tables

**Table:** Amount of Alcohol Use During Specific Social Situations by Ethnicity

	Amount of Alcohol Use by Ethnicity		
	
Social Context	Never (%)	Sometimes (%)	Most of the Time (%)
	**African-American** (*n =* 173)		
Cruising	19.7	52.6	27.7
Group parties	4.0	30.1	65.9
Hanging out with group at night	3.5	38.7	57.8
Hanging out with group during day	11.6	52.0	36.4
School	60.1	12.7	2.3
Before fight	44.5	36.4	19.1
After fight	12.7	39.9	47.4
At home with family	51.4	34.7	13.9
Alone	19.1	60.7	20.2
	**Latino** (*n* = 88)		
Cruising	21.6	59.1	19.3
Group parties	3.4	14.8	80.7
Hanging out with group at night	6.8	37.5	55.7
Hanging out with group during day	20.5	53.4	26.1
School	55.7	29.5	1.1
Before fight	54.5	28.4	15.9
After fight	19.3	35.2	43.2
At home with family	58.0	36.4	4.5
Alone	40.9	43.2	14.8
	**Asian/PI** (*n* = 53)		
Cruising	52.8	34.0	13.2
Group parties	0.0	30.2	69.8
Hanging out with group at night	3.8	45.3	50.9
Hanging out with group during day	28.3	47.2	24.5
School	69.8	26.4	3.8
Before fight	45.3	45.3	9.4
After fight	24.5	49.1	26.4
At home with family	64.2	30.2	5.7
Alone	45.3	49.1	5.7
	**Other** (*n* = 38)		
Cruising	42.1	44.7	13.2
Group parties	0.0	15.8	84.2
Hanging out with group at night	5.3	28.9	63.2
Hanging out with group during day	26.3	55.3	18.4
School	57.9	31.6	2.6
Before fight	42.1	44.7	13.2
After fight	13.2	39.5	47.4
At home with family	55.3	31.6	13.2
Alone	31.6	57.9	10.5

*n* = number of participants within each ethnic category indicated; PI = Pacific Islander.
