# Erratum

**Published:** 2001

A table featuring unpublished data was mistakenly published in the article “Alcohol and Violence in the Lives of Gang Members,” by Geoffrey P. Hunt and Karen Joe Laidler, *Alcohol Research & Health,* Volume 25, Number 1, 2001. The editors regret this error. Readers seeking more information on the data included in the table should contact the authors directly.

